# Fast Clinical Response of Bimekizumab in Nail Psoriasis: A Retrospective Multicenter 36-Week Real-Life Study

**DOI:** 10.3390/ph17101378

**Published:** 2024-10-16

**Authors:** Elena Campione, Fabio Artosi, Ruslana Gaeta Shumak, Alessandro Giunta, Giuseppe Argenziano, Chiara Assorgi, Anna Balato, Nicoletta Bernardini, Alexandra Maria Giovanna Brunasso, Martina Burlando, Giacomo Caldarola, Anna Campanati, Andrea Carugno, Franco Castelli, Andrea Conti, Antonio Costanzo, Aldo Cuccia, Paolo Dapavo, Annunziata Dattola, Clara De Simone, Vito Di Lernia, Valentina Dini, Massimo Donini, Enzo Errichetti, Maria Esposito, Maria Concetta Fargnoli, Antonio Foti, Carmen Fiorella, Luigi Gargiulo, Paolo Gisondi, Claudio Guarneri, Agostina Legori, Serena Lembo, Francesco Loconsole, Piergiorigio Malagoli, Angelo Valerio Marzano, Santo Raffaele Mercuri, Matteo Megna, Giuseppe Micali, Edoardo Mortato, Maria Letizia Musumeci, Alessandra Narcisi, Anna Maria Offidani, Diego Orsini, Giovanni Paolino, Giovanni Pellacani, Ketty Peris, Concetta Potenza, Francesca Prignano, Pietro Quaglino, Simone Ribero, Antonio Giovanni Richetta, Marco Romanelli, Antonio Rossi, Davide Strippoli, Emanuele Trovato, Marina Venturini, Luca Bianchi

**Affiliations:** 1 Dermatology Unit, Department of Systems Medicine, University of Rome Tor Vergata, 00133 Rome, Italy; fabio.artosi994@gmail.com (F.A.); ruslanagaetashumak@gmail.com (R.G.S.); alessandro.giunta@uniroma2.it (A.G.); luca.bianchi@uniroma2.it (L.B.); 2 Dermatology Unit, University of Campania L. Vanvitelli, 80131 Naples, Italy; giuseppe.argenziano@unicampania.it (G.A.); anna.balato@unicampania.it (A.B.); 3 Daniele Innocenzi, Department of Medical-Surgical Sciences and Biotechnologies, Sapienza University Dermatology ASL, 04100 Latina, Italy; chiara.assorgi@uniroma1.it (C.A.); nicoletta.bernardini@libero.it (N.B.); concetta.potenza@uniroma1.it (C.P.); 4 Department of Internal Medicine-Dermatology, Villa Scassi Hospital-ASL3, 16149 Genoa, Italy; alexandra.brunasso@galliera.it; 5 Dermatological Clinic, Department of Clinical and Molecular Sciences, Polytechnic Marche University, 60100 Ancona, Italy; martina.burlando@unige.it (M.B.); info@clinicadermatologicancona.it (A.C.); a.offidani@ospedaliriuniti.marche.it (A.M.O.); 6 Dermatology, Department of Medicine and Translational Surgery, Università Cattolica del Sacro Cuore, 00185 Rome, Italy; giacomo.caldarola@policlinicogemelli.it (G.C.); clara.desimone@unicatt.it (C.D.S.); ketty.peris@unicatt.it (K.P.); 7 Dermatology, Department of Medical and Surgery Sciences, Fondazione Policlinico Universitario A. Gemelli IRCCS, 00168 Rome, Italy; 8 Dermatology Unit, Department of Medicine and Surgery, University of Insubria, 21100 Varese, Italy; acarugno@asst-pg23.it; 9 Section of Dermatology, Koelliker Hospital, 47923 Turin, Italy; franco.castelli@hotmail.it (F.C.); a.conti.dermo@gmail.com (A.C.); 10 Dermatology Unit, IRCCS Humanitas Research Hospital, 10134 Rozzano, Italy; antonio.costanzo@hunimed.eu (A.C.); luigi.gargiulo@humanitas.it (L.G.);; 11 Unit of Dermatology, San Donato Hospital, 52100 Arezzo, Italy; dott.aldocuccia@gmail.com; 12 Second Dermatologic Clinic, Department of Biomedical Science and Human Oncology, University of Turin, 10124 Turin, Italy; paolo.dapavo@gmail.com; 13 Dermatology Unit, Department of Clinical Internal, Anesthesiological and Cardiovascular Science, University of La Sapienza, 00161 Rome, Italy; nancydattola@gmail.com (A.D.); pellacani.giovanni@uniroma1.it (G.P.); antonio.richetta@uniroma1.it (A.G.R.); antoniorossi4019@gmail.com (A.R.); 14 Dermatology Unit, Arcispedale Santa Maria Nuova, Azienda USL-IRCCS di Reggio Emilia, 42123 Reggio Emilia, Italy; vitogiuseppe.dilernia@ausl.re.it; 15 Dermatology Unit, Department of Clinical and Experimental Medicine Ospedale Santa Chiara, 56126 Pisa, Italy; valentinadini74@gmail.com (V.D.); m.romanelli@med.unipi.it (M.R.); 16 Dermatology Unit, Department of Medicine, Hospital S.S. Giovanni e Paolo, AULSS−3-Serenissima, 30122 Venezia, Italy; massimo.donini@aulss3.veneto.it; 17 Institute of Dermatology, Department of Medicine, University of Udine, 33100 Udine, Italy; enzoerri@yahoo.it; 18 Section of Dermatology, Department of Biotechnological and Applied Clinical Science, University of L’Aquila, 67100 L’Aquila, Italy; maria.esposito3@univaq.it (M.E.); mariaconcetta.fargnoli@univaq.it (M.C.F.); 19 Unit of Dermatology, IRCCS Ospedale San Raffaele, 20132 Milan, Italy; antoniofoti06@gmail.com (A.F.); mercuri.santoraffaele@hsr.it (S.R.M.); paolino.giovanni@hsr.it (G.P.); 20 Section of Dermatology, Oncology and Ematology Department Asl Bat, P.O. M.R. Dimiccoli, 70051 Barletta, Italy; carmen.fiorella10@gmail.com; 21 Department of Medicine, Section of Dermatology and Venereology, University of Verona, 37129 Verona, Italy; paolo.gisondi@univr.it; 22 Department of Biomedical and Dental Sciences and Morpho Functional Imaging, Section of Dermatology, University of Messina, 98121 Verona, Italy; claudio.guarneri@polime.it; 23 UO Dermatologia IRCCS Ospedale Galeazzi & Università degli Studi di Milano, 20157 Milan, Italy; info@agostinalegori.it; 24 Department of Medicine, Surgery and Dentistry “Scuola Medica Salernitana” University of Salerno, 84084 Salerno, Italy; slembo@unisa.it; 25 Department of Dermatology, University of Bari, 70121 Bari, Italy; franciscus59@gmail.com (F.L.); edoardo.mortato@gmail.com (E.M.); 26 Department of Dermatology, Dermatology Unit Azienda Ospedaliera San Donato Milanese, 20097 Milan, Italy; dermapier@gmail.com; 27 Dermatology Unit, Fondazione IRCCS Ca’ Granda Ospedale Maggiore Policlinico, 20122 Milan, Italy; angelo.marzano@unimi.it; 28 Department of Pathophysiology and Transplantation, Università degli Studi di Milano, 20122 Milan, Italy; 29 Unit of Dermatologic Clinic, Università Vita-Salute, San Raffaele, 20132 Milan, Italy; 30 Section of Dermatology, Department of Clinical Medicine and Surgery, University of Naples Federico II, 80138 Naple, Italy; carreramat24@libero.it; 31 UOC Dermatologia, University of Catania, PO “G. Rodolico”, AOU Policlinico “G. Rodolico-San Marco”, 95123 Catania, Italy; gimicali1@hotmail.it (G.M.); musumecimarialetizia@gmail.com (M.L.M.); 32 Clinical Dermatology Unit, San Gallicano Dermatological Institute IRCCS, 00167 Rome, Italy; diegorsini@gmail.com; 33 Department of Dermatological Sciences, Dermatology Section, University of Florence, 50121 Florence, Italy; francesca.prignano@unifi.it; 34 Section of Dermatology, Department of Medical Sciences, University of Turin, 10126 Turin, Torino, Italy; pietro.quaglino@unito.it (P.Q.); simone.ribero@unito.it (S.R.); 35 Dermatology Unit, Manzoni Hospital, ASST-Lecco, 23900 Lecco, Italy; davide.strippoli@icloud.com; 36 Dermatology Unit, Department of Medical, Surgical and Neurological Sciences, University of Siena, 53100 Siena, Italy; trovato.ema@gmail.com; 37 Department of Clinical and Experimental Sciences, Section of Dermatology, University of Brescia, 25123 Brescia, Italy; marina.venturini@unibs.it

**Keywords:** bimekizumab, nail psoriasis, interleukin 17, psoriasis, PGA-F, PASI

## Abstract

(1) Background/Objectives: Nail psoriasis (NP) is a chronic and difficult-to-treat disease, which causes significant social stigma and impairs the patients’ quality of life. Moreover, nail psoriasis is a true therapeutic challenge for clinicians. The presence of nail psoriasis can be part of a severe form of psoriasis and can have predictive value for the development of psoriatic arthritis. Our real-world-evidence multicenter study aims to evaluate the efficacy of bimekizumab in nail psoriasis. (2) Methods: A retrospective analysis of a multicenter observational study included 834 patients affected by moderate-to-severe psoriasis, in 33 Dermatologic Units in Italy, treated with bimekizumab from December 2022 to September 2023. Clinimetric assessments were based on Psoriasis Area and Severity Index (PASI), Dermatology Life Quality Index (DLQI), and Physician’s Global Assessment of Fingernail Psoriasis (PGA-F) for the severity of nail psoriasis at 0, 12, 24, and 36 weeks. (3) Results: Psoriatic nail involvement was present in 27.95% of patients. The percentage of patients who achieved a complete clearance of NP in terms of PGA-F 0 was 31.7%, 57%, and 88.5% at week 4, 16, and 36, respectively. PASI 100 was achieved by 32.03% of patients at week 4, by 61.8% at week 16, and by 78.92% of patients at week 36. The mean baseline PASI was 16.24. The mean DLQI values for the entire group of patients at baseline, at week 4, at week 16, and at week 36 were 14.62, 3.02, 0.83, and 0.5, respectively. (4) Conclusions: Therapies that promote the healing of both the skin and nails in a short time can also ensure a lower risk of subsequently developing arthritis which is disabling over time. Bimekizumab proved to be particularly effective to treat NP, with a fast response in terms of complete clearance, with over 88.5% of patients free from NP after 36 weeks. The findings of our real-world study showed that patients with moderate-to-severe PsO and concomitant NP had significantly faster and more substantial improvements in NP up to 36 weeks with respect to previous research findings. Considering the rapid healing of the nail, the dual inhibition of IL17 A and F might have a great value in re-establishing the dysregulation of keratin 17 at the nail level.

## 1. Introduction

Psoriasis is a chronic inflammatory disease that affects up to 3% of the general population. It is more frequent in the white ethnic group and in Northern European countries; it has a lower incidence in Africans, Japanese, and Eskimos, and a very rare incidence in South Americans. Two incidence peaks have been reported, between the second and third, and between the fourth and sixth decades [1,2]. Psoriasis is a systemic inflammatory disease in which the dysregulation of the immune system results in an overexpression of consequently activated T-helper (Th) 17, leading to the uncontrolled proliferation of keratinocytes, acanthosis, neovascularization, and potent skin infiltration by immune cells [3]. The disease is characterized by epidermal hyperproliferation with the formation of erythematous squamous skin plaques that can cover large areas of the body; it is considered a multiorgan disease that requires a multidisciplinary approach and adequate management, taking into account a number of comorbidities [1,2,4]. In the early pathogenic stages of psoriasis, several cell types are present, including plasmacytoid dendritic cells, keratinocytes, natural killer T cells, and macrophages, that secrete cytokines, which activate myeloid dendritic cells [2,5]. Dendritic cells produce TNF-α and IL-23 to promote T cell differentiation toward TH17 cells that produce key psoriatic cytokines IL-17, IFN-γ, and IL-22 [2].

Interleukin 17, in particular, plays a key role in the resulting inflammation and joint injury [3]. It has been initially described as a Th17-produced cytokine, but it is now established that other cell types can also be a source, such as gamma delta T lymphocytes, Mucosal-Associated Invariant T (MAIT) cells, and Innate Lymphoid Cells 3; those cells act as an IL-23-independent source of IL17 in the skin in response to inflammatory stimuli [6]. Interleukin 17, first described by Yao et al. in 1995, is a family of pro-inflammatory cytokines composed of IL-17A, IL-17B, IL-17C, IL-17D, IL-17E, and IL-17F, secreted by T cells, natural killer cells, mast cells, and neutrophils [7]. While IL-17A is more potent, IL-17F is more abundant in skin lesions of psoriasis (by approximately 30-fold), and can drive inflammation independently of IL-17A [7,8,9]. When psoriasis affects visible areas of the body including the face, hands, scalp, and nails, it is strongly associated with physical and quality-of-life impairment, including a negative notable impact on social relationships, mental health, and work activities [10]. The prevalence of nail involvement in psoriatic patients varies between 10% and 90% [9,11,12], with a prevalence of about 32% in children [12]. Approximately 90% of psoriatic patients develop nail psoriasis (NP) during their lifetime and this is not related to gender or age [13,14]. The pathogenesis of NP has not been fully clarified yet, although some peculiar inflammatory cytokines and chemokines seems to be the same as those described in psoriatic skin lesions [15,16,17,18,19]. Nail psoriasis (NP) is often related to long-lasting psoriasis and severity of skin and joint involvement [18,19,20]. Furthermore, psoriatic nail disease may be considered a risk predictor factor for the development of psoriatic arthritis (PSA) and could be considered a form of enthesitis in the early stage of rheumatic disease [19]. Nail lesions, including pitting and onycholysis, occur in approximately 80% to 90% of patients with PsA [21]. The nail bed, nail matrix, hyponychium, and nail folds can be affected by NP. The most observed forms are psoriasis of the nail matrix, the nail bed, and the nail fold [13]. Pitting, leukonychia, red spots of the lunula, transverse furrows (Beau’s lines), and crumbling of the nail plates are the typical signs of nail matrix psoriasis [20]. Oil drop discoloration, splinter hemorrhages involving the distal third of the nail plate, subungual hyperkeratosis, and/or detachment of the nail plate from the nail bed (onycholysis) are characteristic signs of nail bed involvement [13]. Psoriasis of the periungual region is characterized by paronychia [13]. The severity of NP is assessed using the Nail Psoriasis Severity Index (NAPSI) which is a numerical, reproducible, objective, and simple tool. Another tool for a better monitoring of the nail treatment outcome is the Physician’s Global Assessment of Fingernail Psoriasis (PGA-F) [21,22].

PGA-F, developed by Hudgens et al., according to best practices [22], was designed to combine ratings across multiple components of nail severity into one of five distinct classifications ranging from clear to severe. The usefulness of this classification system is that total scores, and changes in scores, are easy to quantify and clinically relevant [22].

Differential diagnosis between onychomycosis and psoriatic nail may be difficult; however, there may even be a coexistence of onychomycosis and NP, and both of them are common disorders in the general population [23,24,25,26,27,28].

Previous findings have shown an increased expression of tumor necrosis factor (TNF)-α, nuclear factor kappa B, IL-6, and IL-8 in psoriasis-affected nails [29,30,31,32]. Several studies have shown a downregulation of IL-10 in psoriatic skin lesions [32]. In contrast, Saulite et al. found an increased IL-10 expression in the affected nail bed, suggesting unique pathways of psoriatic nail disease and toenail as an immune-privileged site [33,34,35].

Nail therapeutic management is based on clinical presentation and patient-related factors. Most patients have mild NP without arthropathic disease or severe skin psoriasis [1]. Topical therapy may be suggested for these patients, while systemic therapy is indicated for patients with severe NP and those with a greater impact on the quality of life or with moderate-to-severe psoriasis. Age, disease burden, comorbidities, individual patient treatment preferences, and treatment risks should be considered to establish the treatment strategy [1]. First-line treatments for few-nail disease (≤3 nails involved) include topicals and intralesional injections. Topical treatments are steroids, vitamin D3 analog calcipotriol or tacalcitol or calcitriol used as monotherapy or combined with corticosteroids, tazarotene, topical calcineurin inhibitors, and 5-fluorouracil [35,36,37,38,39]. 

Regarding biologic agents used to treat NP, most data recorded to date are on adalimumab which is also the only biologic therapy with efficacy data cited in the US Food and Drug Administration’s package insert [35,36,37,38,39,40]. However, many other biologic and systemic therapies have been studied for the treatment of NP, including certolizumab, golimumab, etanercept, infliximab, ustekinumab, guselkumab, tildrakizumab, risankizumab, ixekizumab, secukinumab, brodalumab, and bimekizumab [41]. NP has also been treated with apremilast, a small molecule, that, as a selective inhibitor of phosphodiesterase 4, leads to an increase in cAMP levels, downregulating the expressions of TNFα, IL-17, and IL-23 with an upregulation of the anti-inflammatory IL-10 [41,42].

A systematic literature review gave a strong recommendation for the use of biologic agents including tumor necrosis factor inhibitors (TNFi) and interleukin (IL)-12/23, 17, and 23 inhibitors in patients with NP [38]. Although outcome data are difficult to compare, interleukin (IL)-17 inhibitors may have superior short-term efficacy for NP when compared to IL-23 inhibitors and tumor necrosis factor (TNF)-alpha inhibitors, although long-term efficacy is similar to TNF-alpha inhibitors [42].

Based on the available evidence regarding the role of IL17 in psoriasis and PSA, four therapeutic agents against IL-17A, IL17F, or its receptor have been developed: secukinumab, ixekizumab, brodalumab, and bimekizumab. Significant reductions in NAPSI scores were observed using ixekizumab as early as 2 weeks, up to 20 weeks, and in an open-label extension [43,44,45]. The complete remission of NP was achieved in a high percentage of patients: 43% at week 44 and 51% at week 68 [44]. Brodalumab showed great efficacy in reducing NAPSI in several studies, including three phase 3 trials, ensuring the complete clearance of psoriatic onychopathy at week 52 in almost 64% of affected patients [45]. In a network meta-analysis, brodalumab had the absolute probability of achieving a complete resolution of NP at weeks 24–28 in 37.1% of patients. For bimekizumab, the authors reported an NP complete resolution for 26.7%, 62.1%, and 70.7% of patients at weeks 16, 32, and 48, respectively [46].

The high response rate of NP during anti-IL-17 therapy is also documented by the immunohistochemical evidence of pathogenetically relevant molecules in psoriasis, in particular in NP and PSA [35].

Within this context, cathelicidin (LL-37) is an antimicrobial peptide whose cellular expression levels have been found to be higher in the psoriatic nail bed compared to the control nail bed [35]. IL-17A seems to enhance keratin 17 expression by keratinocytes [47].

A recent study found significantly higher serum IL-17 levels in psoriatic patients with nail involvement compared to those without nail involvement (*p* = 0.002) [48].

Bimekizumab is a humanized IgG1/κ monoclonal antibody that binds selectively and with high affinity to the cytokines IL-17A, IL-17F, and IL-17A/F, blocking their interaction with the IL-17RA receptor complex IL-17RC [49].

Bimekizumab was approved in 2021 by the European Medicine Agency for chronic plaque psoriasis in adult patients eligible for systemic therapy [50].

Real-life data of bimekizumab are quite aligned, showing a fast response in terms of reduction in PASI and DLQI, with approximately 43% of patients able to reach PASI100 after just 4 weeks of therapy and over 70% after 16 weeks, with infrequent adverse reactions of limited severity [51].

In this regard, a multicenter retrospective real-life clinical study is presented to document the efficacy of bimekizumab in NP in a cohort of 834 patients affected by moderate-to-severe psoriasis followed during 36 weeks.

## 2. Results

The clinical and demographic characteristics of our 834 patients recorded at baseline (W0) are shown in Table 1. In particular, 543 were male and 291 were female (Table 2). 

Patients with at least one comorbidity were 464 out of 834 (55.6%); in particular, they were affected by arterial hypertension as the most frequent comorbidity (30.5%), followed by hypercholesterolemia (17%), PsA (13.3%), diabetes (11.2%), heart disease (8.4%), and cancer (3.7%), whereas 23.7% patients were affected by other pathologies (Table 2).

All patients were affected by moderate-to-severe plaque-type psoriasis. Furthermore, 109 patients (13.13%) had concomitant PsA (Table 2).

The involvement of difficult-to-treat sites was also evaluated: 342 (41.0%) patients were affected by scalp psoriasis, 232 (27.95%) had NP, and a further 153 (18.3%) had genital involvement. Furthermore, 11 (1.3%) patients showed palmoplantar psoriasis. 

In addition, stratification was carried out for previous treatments (Table 2): 131 (15.7%) of subjects were naïve to systemic treatment, and 359 (43.05%) were naïve to biologic therapies. A previous biologic therapy failed in 443 patients (53.12%).

In particular, 173 patients (38%) used Adalimumab, 57 (12%) Secukinumab, 53 (11%) Etanercept, 38 (8%) Ustekinumab, 35 (8%) Ixekizumab, 26 (6%) Adalimumab biosimilar, 18 (4%) Brodalumab, 16 (3%) Risankizumab, 13 (3%) Apremilast, 10 (2%) Infliximab, 8 (2%) Guselkumab, 6 (1%) Tildrakizumab, 4 (1%) Etanercept biosimilar, and 4 (1%) Infliximab biosimilar. Some patients had previously undergone more than one treatment with a biologic drug.

The mean baseline PASI was 16.24 (9.03) in the overall patient population (Table 3).

A score of PASI 100 was reached by 32.03% of patients at week 4, by 61.8% at week 16, and by 78.92% of patients at week 36 (Table 4).

Psoriatic nail involvement was present in 27.95% of patients (Table 5), and the clinical and demographic characteristics of this subpopulation of patients are described in Table 6. Patients affected by NP were predominantly male (71.55%), and 84.05% of the entire group had already received previous systemic conventional therapy, whilst 49.14% had one or more previous treatments with a biological drug (Table 7).

The comorbidities found in the NP patient population were mostly similar to those found for the entire patient group (Table 7).

**Figure 1:**
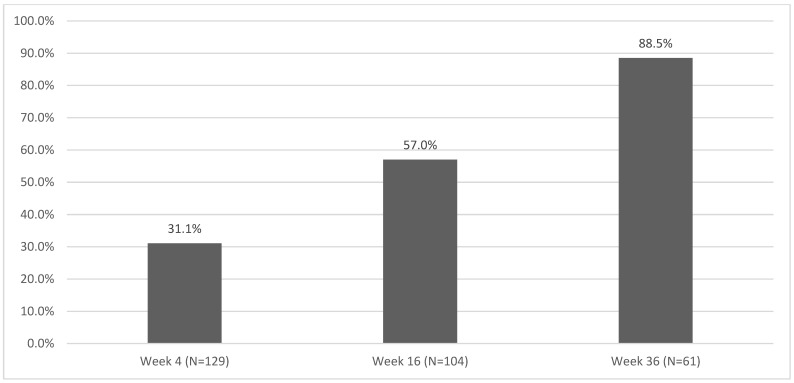
Percentage of patients with PGA-F = 0 at different time points.

Figure 1 shows the percentages of patients who achieved a complete clearance of NP. In total, 31.7%, 57%, and 88.5% of patients achieved PGA-F 0 at week 4, 16, and 36, respectively.

**Figure 2:**
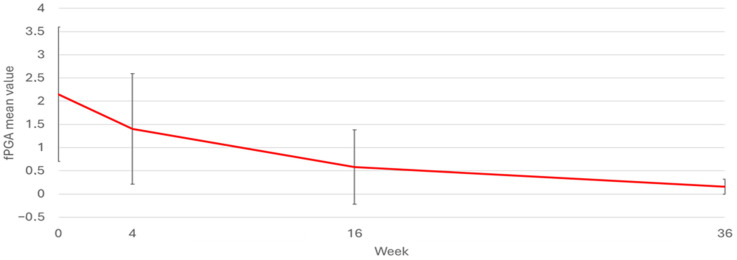
PGA−F mean value over time. Values of PGA−F at baseline, week 4, week 16, and week 36 were 2.15, 1.4, 0.58, and 0.16, respectively.

In Figure 2, it is possible to appreciate the trend in PGA-F at the various time points for the entire group of patients affected by NP. The values of PGA-F at baseline, week 4, week 16, and week 36 were 2.15, 1.4, 0.58, and 0.16, respectively. The mean percentage reduction in PGA-F from baseline to week 36 was 92.5% (*p*-value < 0.0001).

**Figure 3:**
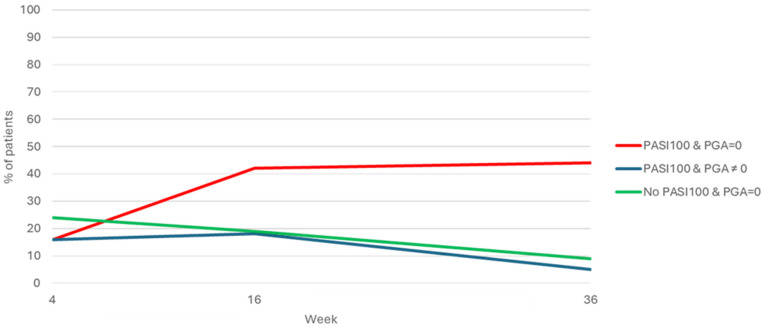
Percentage of patients with complete clearance of NP and PSO assessed by PGA-F = 0 and PASI100 at different time points.

In the subgroup of patients with nail involvement, the percentage of patients who achieved complete skin and nail clearance was reported at different time points (Figure 3). At W4, the population reaching PASI 100 and PGA-F = 0, PASI 100 and PGA-F ≠ 0, and no-PASI 100 and PGA-F = 0 was 12.21%, 12.21%, and 18.32%, respectively. At W16, the population reaching PASI 100 and PGA-F = 0, PASI 100 and PGA-F ≠ 0, and no-PASI 100 and PGA-F = 0 was 39.25%, 16.82%, and 17.76%, respectively. At W36, the population reaching PASI 100 and PGA-F = 0, PASI 100 and PGA-F ≠ 0, and no-PASI 100 and PGA-F = 0 was 73.33%, 8.33%, and 15%, respectively (Figure 3). Figure 1 shows the group that did not achieve complete clearance at the various control time points, corresponding to the smallest one.

**Figure 4:**
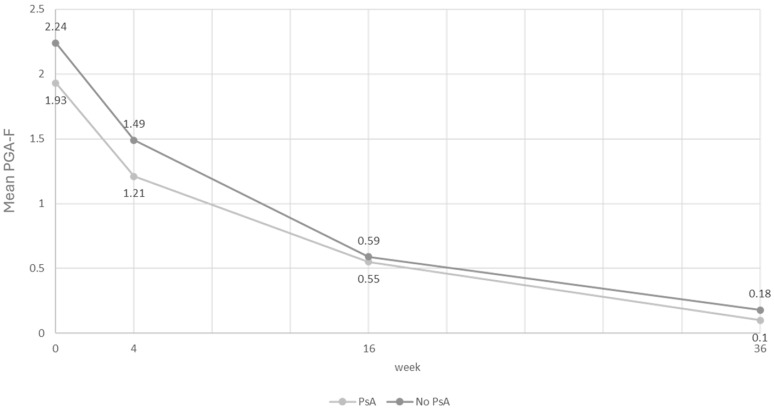
Graphical trend in PGA-F stratified by presence or absence of PSA in patients with NP.

Among patients suffering from NP, those also suffering from PSA showed slightly lower mean baseline PGA-F values than patients without PSA (Table 8). The difference in the reduction in PGA-F in the two subgroups of patients did not show any statistical significance, although there was an appreciable reduction at W36 of 2.27 times and 2.4 times the average value of PGA-F in the population without PsA and in that with PsA, respectively (Table 8). The trend at the various time points is comparable between the two groups, showing a rapid and parallel reduction in PGA-F values (Figure 4).

Wilcoxon *p*-value between PsA vs. no PsA. Change at week 4 vs. baseline: 0.1375. Change at week 16 vs. baseline: 0.7175. Change at week 36 vs. baseline: 0.7939.

**Figure 5:**
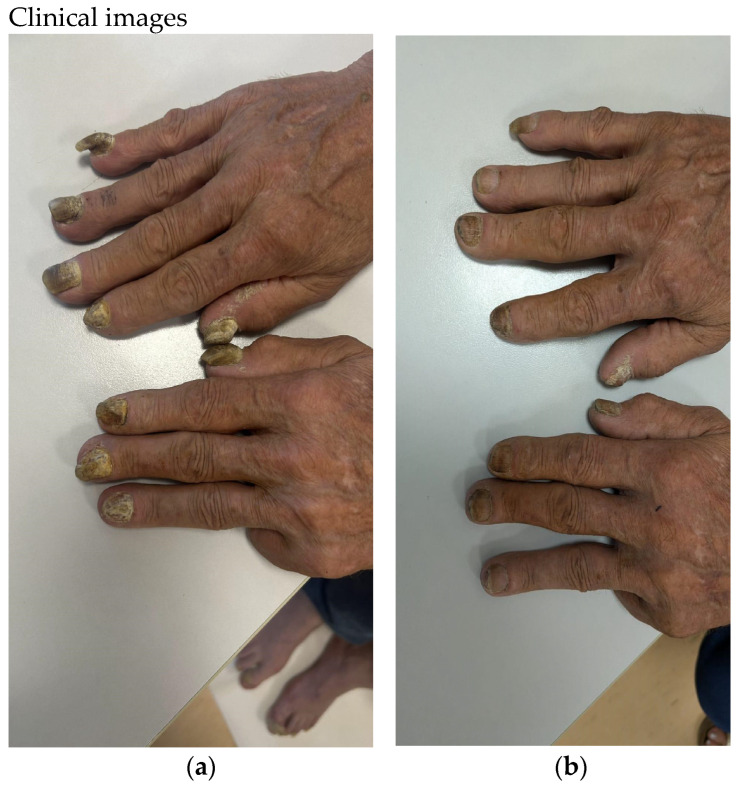
(**a**) Case 1—Nail psoriasis in male patient at baseline, PGA-F: 4. (**b**) After 36 weeks of treatment with bimekizumab, PGA-F: 2.

**Figure 6:**
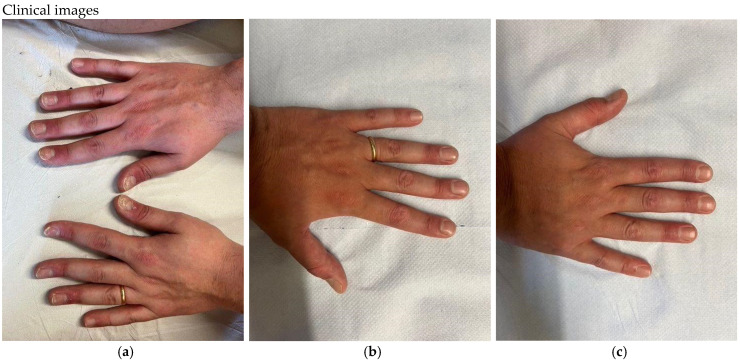
(**a**) Nail psoriasis in female at baseline. PGA-F: 3. (**b**,**c**) After 36 weeks of bimekizumab treatment- PGA-F: 0.

The mean DLQI values for the entire group of patients at baseline, at week 4, at week 16, and at week 36 were 14.62, 3.02, 0.83, and 0.5, respectively. The mean DLQI reduction from baseline to week 36 was 96.6% (Table 9). Two clinical cases of nail psoriasis can be seen in Figure 5 and Figure 6.

The mean DLQI values for the group of NP-affected patients were 15.33, 3.70, 1.08, and 0.57 at baseline, week 4, week 16, and week 36, respectively (Table 10).

## 3. Materials and Methods

A retrospective multicenter clinical study was conducted in 33 Italian Dermatologic Units. We collected data on 834 patients aged ≥ 18 years with moderate-to-severe plaque psoriasis, treated with bimekizumab, a new inhibitor of IL17A and IL17F. The recommended bimekizumab regimen consisted in two subcutaneous injections of 160 mg administered at week 0, 4, 8, and 16 and then every 8 weeks. Patients were monitored for 36 weeks from baseline (visit 1, day 0 prior to the first dose). The study was conducted in accordance with Good Clinical Practice, the applicable regulatory requirements, and the Declaration of Helsinki. All patients provided written informed consent prior to participating in the study.

### 3.1. Assessments and Outcomes

Patient demographics and psoriasis disease characteristics were collected at the baseline visit. The assessment criteria used to evaluate the severity of skin disease were the Psoriasis Area and Severity Index (PASI) and the Dermatology Life Quality Index (DLQI). Nail psoriasis was evaluated by measuring PGA-F. The score includes the assessment of symptoms and scoring table to individually rate the level of involvement of the nail bed and nail matrix using predefined categories of “clear” (0), “minimal” (1), “mild” (2), “moderate” (3), and “severe” (4). Each grade was supplemented by a general description of the criteria needed to qualify for assignment. Patients whose signs and symptoms were intermediate between two grades were assigned the higher of the two. The score from the nail area (matrix or nail bed) with the highest level of involvement was then selected to generate a total global assessment score [21].

### 3.2. Statistical Analysis

Quantitative variables were described using mean, standard deviation (SD), median, and minimum and maximum values, while qualitative variables were reported using absolute and relative frequencies.

Missing values were neither replaced nor considered for the analysis of that variable. The assessment of PASI values was calculated at baseline. The proportion of patients reaching PASI100 and PGA-F equal to zero were calculated at week 4, 16, and 36.

A subgroup of patients with nail psoriasis were also described using the same methods described above. Among these patients were a class of patients with PsA and diabetes. Comparison between cohorts was carried out using the Wilcoxon test. All *p*-values < 0.05 were considered significant.

## 4. Discussion

Nail psoriasis is a chronic and difficult-to-treat disease, which causes significant social stigma, impairs the patients’ quality of life, and is a true therapeutic challenge for clinicians. The presence of NP could be associated with a more severe form of psoriasis but also has a predictive value for the development of PsA. The nail bed is close to the periosteum of the distal phalanx, and the nail matrix lies near the distal interphalangeal joint and the insertion of the digital extensor tendon. This anatomic structure favors the common association of NP with distal interphalangeal joint arthritis and enthesitis [17,19]. An early diagnosis of enthesitis and distal interphalangeal joint arthritis by radiological imaging (ultrasonography or magnetic resonance imaging) allows clinicians to establish the appropriate treatment, and thus it enables optimal outcomes, improves prognosis, and prevents deformities [17,51]. Therefore, an early treatment is mandatory since it is decisive in blocking the onset of PsA; thus, biologics are a promising option for NP. The data about network meta-analyses (NMAs) of randomized clinical trials have documented the relative efficacy of different biologics [11,46].

The upregulation of several cytokines and kinases has been implicated in the pathogenesis of psoriasis, including tumor necrosis factor-α (TNF-α), IL-17A, IL-17F, and IL-23. These inflammatory mediators are crucial in the development and maintenance of the changes in skin observed in psoriasis.

The IL-17 family is one of the main effectors for the development of cutaneous and nail psoriatic lesions. Interleukin-17F shares 50% homology with IL-17A. It also causes the release of pro-inflammatory cytokines and mobilizes neutrophils [52,53]. Therefore, in psoriasis lesions, the pro-inflammatory effects on keratinocytes and neutrophils are due to both members of the IL-17 family.

Based on the available evidence regarding the role of IL17 in psoriasis and PSA, four therapeutic agents against IL-17A, IL17F or its receptor have been developed: secukinumab, ixekizumab, brodalumab, and bimekizumab. Significant reductions in NAPSI scores were observed using ixekizumab as early as 2 weeks, up to 20 weeks, and in an open-label extension [43,44,45]. The complete remission of NP was achieved in a high percentage of patients: 43 % at week 44 and 51% at week 68 [44]. Brodalumab showed great efficacy in reducing NAPSI in several studies, including three phase 3 trials, ensuring the complete clearance of psoriatic onychopathy at week 52 in almost 64% of affected patients [45]. In a network meta-analysis, brodalumab had the absolute probability of achieving a complete resolution of NP at weeks 24–28 in 37.1% of patients. For bimekizumab, the authors reported a NP complete resolution for 26.7%, 62.1%, and 70.7% of patients at week 16, 32, and 48, respectively [46]. Bimekizumab showed superior efficacy respect to comparators in pivotal phase 3 studies with the same safety profile. The PsO clinical trial program included four phase 3 studies enrolling a total of 2223 patients with moderate-to-severe plaque psoriasis: BE VIVID (compared to placebo and ustekinumab), BE READY (compared to placebo), BE SURE (compared to adalimumab), and BE RADIANT (compared to secukinumab). The primary endpoints were superior to placebo (in the BE VIVID and BE READY studies), to adalimumab (BE SURE study) and to secukinumab (BE RADIANT study); bimekizumab was also superior to ustekinumab in secondary endpoints classified in BE VIVID [46]. A BE VIVID post hoc subanalysis showed the higher efficacy of bimekizumab on NP, with a modified nail psoriasis index score (mNAPSI) of 0 achieved by 54% of patients after 52 weeks of treatment [54].

In this multicenter clinical study, 28% of patients were affected by NP, in line with the evidence in the literature, since it affects a percentage of 10–82% of psoriasis patients [55,56]. Furthermore, NP was found more frequently in men (71.55%) than in women (28.45%).

Our results highlighted a higher PASI decrease during treatment with bimekizumab than previously reported [57], with PASI 100 response in 62% of patients after 16 weeks and 79% after 36 weeks.

Bimekizumab also proved to be particularly effective to treat NP, with a fast response in terms of complete clearance, with almost 32% of patients achieving PGA-F 0 after just 4 weeks. The PGA-F score, in fact, considers not only nail bed alterations with a growth slower than 4 weeks, but also the presence of splinter hemorrhages, nail bed erythema, and subungual hyperkeratosis. It should be noted that in our cohort, bimekizumab had superior efficacy when compared to other anti-IL-17 drugs such as brodalumab and ixekizumab [44], demonstrating a complete response of NP (PGA-F 0) in 88.5% of patients after 36 weeks.

In their NMA, Egberg et al. considered a score of zero on the NAPSI, modified NAPSI, or Physician’s Global Assessment of Fingernails (PGA-F) as an outcome of complete clinical resolution for NP [46]. PGA-F is a rapid, valid, and reliable measure of psoriatic nail disease compared to other NP scoring systems. PGA-F was developed according to best practices for instrument development described by the FDA, and is the only system with established content validity [21]. The PGA-F total score accurately reflects the overall severity of NP [21]. Our data underlined a great outcome, with a mean reduction in PGA-F during follow-up of 92.5%, despite 56.9% of patients being bio-experienced, so at risk of higher rates of primary and secondary therapeutic ineffectiveness [58]. Both skin and nails achieved a quick clearance. Indeed, in patients with both PsO and NP, the simultaneous achievement of skin and nail clearance was the most observed event, since these patients represented the largest group at week 16 (39.25%) and week 36 (73.33%). On the other hand, the two populations that separately achieved PASI100 or PGA-F 0 tended to progressively decrease, reaching a minimum percentage of enrolled patients at week 36 (8.33% and 15%; Figure 3). Elisabeth Riedl et al. also confirmed that patients with PsO and concomitant NP have significantly better and faster improvements in their NP when treated with anti-IL-17 drugs, compared to other classes of biological drugs [59]. Therefore, our data confirm the strong rationale of the use of bimekizumab in patients with PsO and concomitant NP.

No significant difference was recorded in the trend at different time points of NP when patients were divided by presence or absence of PSA, which leads us to also hypothesize a simultaneous action on the latter, since the two entities are intimately related both at the pathogenetic level and for clinical progression [60]. However, data related to the response of PSA to bimekizumab were not the aim of this paper and will be the subject of future publications.

Furthermore, the rapid response to the drug in terms of the complete healing of the skin and nails was reflected in the quality of life of the patients, with 96.6% reduction in DLQI from baseline.

## 5. Conclusions

The results of our real-world study showed that patients with moderate-to-severe PsO and concomitant NP had significantly faster and more substantial improvements in NP up to 36 weeks with respect to previous findings. Considering the complete healing of the nail, the dual inhibition of IL17 A and F probably has a greater value in re-establishing the dysregulation of keratin 17 at the nail level. The use of bimekizumab in a greater number of subjects also suffering from PsA will give us even more indications in clinical practice to choose the most suitable drugs for our patients.

## Figures and Tables

**Table 1:** Demographic and clinical characteristic of enrolled patients.

Variable	N	Mean	SD	Median	Minimum	Maximum
Age at BKZ first dose (years)	834	50.13	14.77	51.00	18.00	87.00
Weight (kg)	792	81.01	17.63	80.00	46.00	178.00
Height (cm)	791	171.93	10.04	173.00	72.00	201.00
BMI	791	27.49	6.99	26.31	16.90	125.00
Time from PSO diagnosis (years)	697	14.83	12.57	12.00	0.00	57.00

**Table 2:** Gender distribution and other clinical characteristics of enrolled patients.

Gender	Frequency	Percent
Female	291	34.89
Male	543	65.11
Covid-19	**Frequency**	**Percent**
NO	557	66.79
YES	277	33.21
Naive to systemic treatment	**Frequency**	**Percent**
NO	703	84.29
YES	131	15.71
Naive to biologic therapies	**Frequency**	**Percent**
NO	385	46.1%
YES	359	43.05%
Undefined	32	3.84%
Arthritis	**Frequency**	**Percent**
NO	693	83.49
YES	109	13.13
Undefined	28	3.37
Other comorbidities	**Frequency**	**Percent**
None	370	44.36
At least one comorbidity	464	55.64
Cardiovascular disease	70	8.4
Diabetes	93	11.2
Hypertension	254	30.5
Hyperlipidemia	142	17.0
Neoplasias	31	3.7
Other	198	23.7

**Table 3:** PASI and DLQI at baseline.

Variable	n	Mean	SD	Median
**PASI**	834	16.24	9.03	15.00
**DLQI**	830	14.62	8.81	15.00

**Table 4:** PASI100 at each time point.

PASI100	Frequency	Percent
**Week 4 (n = 512)**	164	32.03
**Week 16 (n = 411)**	254	61.80
**Week 36 (n = 223)**	176	78.92

**Table 5:** Presence of nail involvement at baseline.

Nail Involvement	Frequency	Percent
NO	579	69.76
YES	232	27.95
Undefined	19	2.29
**Frequency Missing = 4**		

**Table 6:** Demographic data and baseline characteristics in patients with nail involvement at baseline.

Variable	n	Mean	SD	Median	Minimum	Maximum
Age at BKZ first dose (years)	232	51.52	14.21	53.00	19.00	87.00
Weight (kg)	226	82.35	16.77	80.00	46.00	178.00
Height (cm)	225	173.13	8.48	175.00	148.00	193.00
BMI	225	27.44	5.02	26.56	17.63	54.94
Time from diagnosis (years)	183	14.28	11.86	12.00	0.00	48.00

**Table 7:** Gender distribution and other clinical characteristics of patients with nail involvement at baseline.

	Frequency	Percent
Female	66	28.45
Male	166	71.55
Covid-19	**Frequency**	**Percent**
NO	135	58.19
YES	97	41.81
Systemic therapy	**Frequency**	**Percent**
NO	195	84.05
YES	37	15.95
Bio-naive	**Frequency**	**Percent**
**NO**	105	49.53
**YES**	107	50.47
**Comorbidities**	**Frequency**	**Percent**
**None**	93	40.1%
**Comorbidities ≥ 1**	139	59.9%
Cardiovascular diseases	19	8.2%
Diabetes	28	12.1%
Hypertension	81	34.9%
Hyperlipidemia	45	19.4%
Neoplasias	10	4.3%
Other	71	30.6%

**Table 8:** PGA-F stratified by PsA presence/absence at baseline.

PsA	Variable: PGA-F	N	Mean	SD	Median	Minimum	Maximum
NO	Baseline	158	2.24	1.43	3.00	0.00	4.00
	Week 4	101	1.49	1.22	2.00	0.00	4.00
	Week 16	83	0.59	0.84	0.00	0.00	4.00
	Week 36	49	0.18	0.63	0.00	0.00	4.00
	Change w4 vs. baseline	99	−0.91	1.09	−1.00	−4.00	1.00
	Change w16 vs. baseline	81	−1.72	1.25	−2.00	−4.00	2.00
	Change w36 vs. baseline	49	−2.27	1.25	−2.00	−4	0.00
YES	Baseline	43	1.93	1.5	2.00	0.00	4.00
	Week 4	28	1.21	1.07	1.00	0.00	3.00
	Week 16	22	0.55	0.67	0.00	0.00	2.00
	Week 36	10	0.10	0.32	0.00	0.00	1.00
	Change w4 vs. baseline	27	−1.33	1.24	−1.00	−4.00	0.00
	Change w16 vs. baseline	21	−1.81	1.17	−2.00	−3.00	0.00
	Change w36 vs. baseline	10	−2.4	1.07	−3.00	−4.00	−1.00

**Table 9:** Evaluation of DLQI at baseline, and at weeks 4, 16, 36.

Variable	N°	Mean	Std Dev	Median	Minimum	Maximum
**Baseline**	830	14.62	8.81	15.00	0.00	30.00
**Week 4**	512	3.02	4.06	2.00	0.00	23.00
**Week 16**	411	0.83	1.87	0.00	0.00	15.00
**Week 36**	225	0.50	1.66	0.00	0.00	15.00

**Table 10:** DLQI—Nail psoriasis population.

Variable	N°	Mean	Std Dev	Median	Minimum	Maximum
**Baseline**	210	15.33	9.19	17.00	0.00	30.00
**Week 4**	136	3.70	4.74	2.00	0.00	23.00
**Week 16**	108	1.08	2.23	0.00	0.00	11.00
**Week 36**	61	0.57	1.62	0.00	0.00	8.00

## Data Availability

Data is contained within the article.
